# Experimental study of factors influencing observers’ perceptions and reactions to sexual harassment in Chinese university students

**DOI:** 10.3389/fpsyg.2025.1525006

**Published:** 2025-08-14

**Authors:** Zien Huang, Jialuo Lai, Fei Xin

**Affiliations:** School of Psychology, Shenzhen University, Shenzhen, China

**Keywords:** sexual harassment, empathy, sexism, feminism, victim blaming

## Abstract

**Introduction:**

Sexual harassment, a pervasive form of gender-based violence, inflicts profound adverse effects on survivors. Observers’ perceptions and responses critically shape subsequent attitudes and behaviors. A systematic comprehension of the determinants that influence observers’ perception of harassment, as well as tendencies for victim-blaming and sympathy, is crucial for devising efficacious intervention strategies. However, existing research on these factors is fragmented, and studies within the Chinese context are notably scarce.

**Methods:**

This study employed an experimental approach to examine factors influencing observers’ perceptions and reactions to sexual harassment in China, including the type of harassment (gender harassment, unwanted sexual attention, or sexual coercion), observer gender, and observer characteristics (empathy, moral sensitivity, willingness to engage in feminist behaviors, sexism, sexual narcissism, sexual harassment myths, and tolerance of sexual harassment).

**Results:**

The type of harassment and gender influenced observers’ perception of harassment, emotional response, level of blame attributed to the victims, and sympathy toward the victims’ suffering. Observer characteristics further modulated perceptions and reactions, bifurcating into two distinct systems. Observers with higher empathy, moral sensitivity, and feminist action readiness (the positive system) exhibited increased sensitivity, emotional connection, sympathy, and reduced victim-blaming. Conversely, those with higher sexism, sexual narcissism, sexual harassment myth endorsement, and harassment tolerance (the negative system) demonstrated diminished sensitivity, emotional engagement, sympathy, and augmented victim-blaming.

**Discussion:**

The findings indicate that effective interventions to prevent and reduce sexual harassment should address the underlying beliefs and values shaping how individuals perceive and respond to such incidents.

## Introduction

Sexual harassment is a prevalent form of gender-based violence that inflicts significant negative effects on survivors, such as psychological distress, work-related problems, and educational difficulties (Rospenda et al., 2023; Blindow et al., 2024). Sexual harassment research encompasses several crucial concepts, such as perceptions of sexual harassment, victim blaming, and victim sympathy. These concepts, alongside gender-related notions, play a significant role in comprehending the factors influencing sexual harassment and devising effective intervention strategies.

Perceptions of sexual harassment hold significance in ascertaining whether misconduct has taken place and whether allegations of harassment carry legal or punitive weight (Rotundo et al., 2001). These perceptions also shape observers’ responses to such situations – whether they choose to report it, support the victim, confront the perpetrator, or intervene as a bystander. Moreover, these perceptions affect how survivors cope with their experiences and how perpetrators justify their actions. The emotional responses of observers prompted by incidents of sexual harassment also hold substantial influence over their attitudes, judgments, and willingness to intervene. Some observers may experience feelings of anger and outrage in response to witnessing sexual harassment. Those who experience pronounced emotional reactions (compared to those who do not) are more likely to take immediate intervention action (Bowes-Sperry and O’Leary-Kelly, 2005). Moreover, higher levels of negative emotions elicited by witnessing injustice were associated with less victim derogation (Ash and Yoon, 2020). Victim blaming occurs when individuals attribute responsibility or culpability to the victim of sexual harassment for the incident or its aftermath, instead of holding the perpetrator accountable (De Judicibus and McCabe, 2001; Taylor, 2020). This demeanor shifts attention from the wrongdoings of the harasser to the victim’s conduct, appearance, or choices, insinuating that they somehow invited or provoked the harassment. Victim blaming generates harmful and unfair judgments of the victim and may deter them from reporting the harassment or seeking assistance. Conversely, sympathy for the victim denotes the sentiments of compassion, understanding, or support directed at survivors of sexual harassment. Understanding and addressing victim blaming, while concurrently promoting victim sympathy, are critical in combating sexual harassment and nurturing a culture of respect, equality, and support for all individuals.

### Factors affecting observers’ perception of sexual harassment, victim blaming, and victim sympathy

A multitude of factors can influence observers’ perceptions of sexual harassment, as well as their propensity to assign blame or sympathy toward the victim or perpetrator. Certain factors may positively affect observers’ perceptions and responses, while others may negatively impact them.

#### Sexual harassment categories

Sexual harassment is a broad term, including many types of unwelcome verbal and physical sexual attention. According to the tripartite model, sexual harassment has three categories: gender harassment, unwanted sexual attention, and sexual coercion (Fitzgerald et al., 1995). Gender harassment encompasses a broad range of verbal and non-verbal behaviors not aimed at sexual cooperation but that convey insulting, hostile, and degrading attitudes about women (Fitzgerald et al., 1995). Examples include anti-female jokes, comments questioning women’s suitability for management positions, and the use of crude terms to denigrate women (e.g., referring to a coworker as a “dumb slut”). Unwanted sexual attention involves unwelcome and offensive expressions of romantic or sexual interest that are not reciprocated by the recipient (e.g., unwanted touching, pressuring for dates, or sexual behavior). The third category, sexual coercion, entails the use of bribes or threats to make the victim’s employment conditions contingent on engaging in sexual activities (e.g., offering a promotion in exchange for sexual favors or threatening termination unless sexual demands are met). Some studies have shown that gender harassment is perceived less as sexual harassment in comparison to unwanted sexual attention (Saunders and Senn, 2009; Herrera Enríquez et al., 2018). Nevertheless, research remains scant regarding the impact of distinct types of sexual harassment on observers’ perceptions, emotional reactions, and the extent of victim-blaming and sympathy.

#### Observer gender

It has been consistently shown that men and women differ in how they perceive sexual harassment. Specifically, men tend to downplay, rationalize, or deny the occurrence and severity of sexual harassment more than women do (Leigh et al., 2021; Rothgerber et al., 2021; Harush et al., 2023). Moreover, male observers tend to assign greater blame and exhibit less sympathy toward victims of sexual violence compared to female observers (De Judicibus and McCabe, 2001; Osman, 2011; Van der Bruggen and Grubb, 2014). The emotional responses of observers to harassment incidents may also affect their perceptions of sexual harassment, as well as their tendencies for victim-blaming and sympathy. To our knowledge, current studies lack an exploration of how these emotional reactions to sexual harassment vary by gender and the types of harassment. This study seeks to bridge this gap by examining the effects of gender and different categories of sexual harassment on observers’ emotional reactions, specifically in terms of emotional valence and arousal.

A limited number of studies have examined the variation of gender differences by the type of sexual harassment, yet they have obtained inconsistent results (Rotundo et al., 2001; Rothgerber et al., 2021). A meta-analysis by Rotundo et al. (2001) revealed that gender difference was larger for less extreme and more ambiguous behaviors, such as derogatory attitudes and dating pressure than for sexual propositions and sexual coercion. However, Rothgerber et al. (2021) reported opposite results, suggesting that gender differences were smaller for less severe sexual harassment, such as derogatory attitudes and non-sexual physical contact. Rothgerber et al. (2021) also examined the gender differences in terms of three dimensions of sexual harassment (gender harassment vs. unwanted sexual attention vs. sexual coercion). However, they did not find a significant interaction between participant gender and the sexual harassment dimension. Given these inconsistent findings and the lack of research within the Chinese context, it is imperative to reassess how perceptions of sexual harassment and reactions vary by observer gender and harassment type.

#### Observer characteristics

Research on observer characteristics and their impact on perceptions of and reactions to sexual violence remains fragmented and lacks an integrative framework to connect these variables. The characteristics that have received more comprehensive attention include gender (Rothgerber et al., 2021; Harush et al., 2023), sexist attitudes (De Judicibus and McCabe, 2001; Gutierrez and Leaper, 2024), and sexual harassment/rape myths acceptance (Van der Bruggen and Grubb, 2014; Li and Zheng, 2022). Drawing from extant research, it is evident that certain observer characteristics may exert a positive influence on perceptions and reactions to sexual harassment, while others may have a negative effect.

Characteristics such as empathy, moral sensitivity, favorable views of feminism and the women’s movement, and willingness to engage in feminist behaviors (WEFB) may cultivate a more supportive and empathetic environment for victims of sexual harassment, simultaneously contesting attitudes that blame the victim. These attributes can inspire observers to take a more active and compassionate approach in addressing and responding to sexual harassment incidents.

Empathy is the ability to share, understand, and respond with care to the affective states of others. Observers who possess higher levels of empathy for the victim of sexual harassment may be more likely to perceive the behavior as harmful and unacceptable, to blame the perpetrator for their actions, and to support the victim. On the contrary, observers who have higher empathy for the perpetrator of sexual harassment may be more likely to perceive the behavior as normal or unintentional, to blame the victim for their situation, and to justify the perpetrator (Bongiorno et al., 2019). Moral sensitivity is the ability to recognize and interpret moral issues in social situations, and is a necessary component of moral behavior (Jordan, 2007; Zhang and Xiang, 2022). It includes dimensions such as interpreting others’ reactions and feelings, having empathy and role-taking ability, understanding how one’s actions can affect the welfare of the self and others, and making inferences from others’ behavior and responding appropriately to their reactions (Jordan, 2007). Sexual harassment may occur less frequently if actors are encouraged to regard sexually harassing behavior as involving a moral component (O’Leary-Kelly and Bowes-Sperry, 2001). Therefore, observers with higher moral sensitivity are more likely to recognize and oppose sexual harassment (Goodwin et al., 2020). However, there appears to be a lack of research investigating the effect of moral sensitivity on the perception and response to sexual violence.

Attitudes toward feminism and the women’s movement (Fassinger, 1994) and WEFB (Redford et al., 2018) may also affect how observers perceive sexual harassment, empathize with the victim, and blame the perpetrator or the victim. One study conducted in China found that active commitment to feminism was positively correlated with women’s perception of sexual harassment, while passive acceptance of traditional gender roles was positively correlated with tolerance of sexual harassment (Shi and Zheng, 2020). In addition, the types of targets and types of sexual harassment moderated the relationship between feminist Active Commitment and the perception of sexual harassment (Shi and Zheng, 2021). However, the studies described above included only Chinese women as participants, thereby failing to consider gender differences.

Conversely, sexual harassment myths, tolerance of sexual harassment, sexism, and sexual narcissism can negatively impact observers’ reactions to sexual harassment, potentially diminishing empathetic responses toward victims, fostering a tendency to blame victims, and trivializing the gravity and resultant harm of these incidents. Proactively addressing and challenging these detrimental beliefs and attitudes is crucial for fostering an environment that is more supportive and respectful toward victims of sexual harassment.

Acceptance and tolerance of sexual harassment refer to the degree to which observers explicitly or implicitly approve, excuse, or ignore sexually harassing behaviors (Russell and Trigg, 2004; Shi and Zheng, 2020). Studies indicate that men exhibit greater tolerance for sexual harassment than women (Russell and Trigg, 2004). Observers with high tolerance of sexual harassment may underestimate or disregard the severity or harm of harassment incidents (LeMaire et al., 2016), and are more likely to embrace rape myths (LeMaire et al., 2016). Sexual harassment myths are false beliefs that deny, justify, or trivialize sexual harassment against women (Lonsway et al., 2008), which is closely associated with rape myths. Some examples of sexual harassment myths are: “She asked for it,” “He didn’t mean to,” and “She lied.” Myth-accepting observers are more likely to perceive sexual harassment as a harmless or humorous behavior, to blame the victim for causing or consenting to the harassment, and to empathize with the perpetrator for being innocent or misunderstood (Rollero and Tartaglia, 2019; Long and Herr, 2022). Research has shown that acceptance of sexual harassment myths is influenced by factors such as sexism, sexual narcissism, gender, social identity, and situational context (Rollero and Tartaglia, 2019; Long and Herr, 2022).

Sexism is the belief that one sex is superior to another, and that this justifies discrimination or prejudice based on sex. Sexism can take two forms: hostile sexism, which is overtly negative and derogatory toward women, and benevolent sexism, which is covertly positive but patronizing and protective toward women (Glick and Fiske, 1996). Observers with sexist beliefs may downplay the seriousness of the harassment or attribute it to harmless flirtation rather than recognizing it as inappropriate and harmful behavior (Wiener et al., 1997). Sexism has been shown to associate with higher acceptance of rape myths and sexual harassment myths (De Judicibus and McCabe, 2001; Rollero and Tartaglia, 2019). Sexism can also affect how observers assign blame or empathy to the victim of sexual harassment, with sexist observers being more likely to blame the victim for provoking or inviting the harassment (De Judicibus and McCabe, 2001; Rollero and Tartaglia, 2019).

Sexual narcissism is a personality trait that reflects an inflated sense of sexual entitlement, ability, and performance, as well as a lack of sexual empathy and intimacy (Widman and McNulty, 2010). Sexual narcissism has been shown to be related to higher acceptance of rape myths, as well as higher likelihood of engaging in sexual aggression (Widman and McNulty, 2010; Long and Herr, 2022). Sexual narcissism may also affect how observers perceive sexual harassment, victim blaming and victim sympathy. Observers with high levels of sexual narcissism may regard sexual harassment as normal or flattering, hold the victim responsible for rejecting or resisting the perpetrator’s advances, and sympathize with the perpetrator for pursuing their sexual goals. However, no studies have directly examined this hypothesis.

### The current study

It is imperative to discern the factors that influence observers’ perception of sexual harassment, emotional reactions, as well as tendencies for victim-blaming and sympathy. However, the existing body of research in this domain is fragmented, with most studies examining isolated factors, such as gender differences or singular aspect of observers’ characteristics. Despite the growing awareness of sexual harassment as a prevalent issue in China (Wang et al., 2022; Xu and Zhang, 2022), research on this topic remains scarce. To our knowledge, only two studies have specifically focused on the perception of sexual harassment and its influencing factors in the country (Shi and Zheng, 2021; Mou et al., 2022). Both studies exclusively included female participants. Shi and Zheng (2021) explored the relationship between feminist Active Commitment and the perception of sexual harassment among Chinese working women, discovering a positive correlation between higher levels of Active Commitment and the recognition of both types of sexual harassment. Mou et al. (2022) assessed the general attitudes and perceptions of Chinese female college students toward sexual harassment and the #metoo campaign. The study also investigated the influence of traditional gender role values, hostile sexism, and benevolent sexism on tolerance of sexual harassment and endorsement of the #metoo social media campaign.

The topic of sexual harassment has traditionally been considered sensitive and taboo in Chinese society, which might have contributed to the lack of extensive research on this subject. Cultural factors are known to shape perceptions and responses to sexual harassment (Kennedy and Gorzalka, 2002; Dworkin and Weaver, 2021). The unique cultural context, social norms, gender dynamics, power structures, and traditional beliefs about gender roles in China may influence perceptions and reactions to sexual harassment in diverse ways. Thus, it is imperative to conduct an exhaustive investigation into the myriad factors—both advantageous and detrimental–that influence observers’ perceptions and reactions to sexual harassment within the Chinese context. Against this background, this study aimed to construct an integrated understanding by investigating the impact of different types of sexual harassment (gender harassment, unwanted sexual attention, and sexual coercion), as well as observers’ gender and characteristics, on their perceptions and reactions to sexual harassment in China.

### Hypotheses

H1. There is an anticipated gender difference in perceptions and reactions to sexual harassment. Male observers will downplay the seriousness of sexual harassment incidents more than female observers. Female observers are expected to exhibit stronger emotional responses and greater empathy toward victims, whereas male observers are likely to attribute more blame to the victims.

H2. The type of sexual harassment incident is predicted to influence observers’ perceptions of harassment, emotional responses, victim-blaming tendencies, and levels of sympathy for the victim. More specifically, gender harassment is anticipated to be perceived as less severe compared to unwanted sexual attention and sexual coercion.

H3. Certain individual characteristics of observers, such as empathy ability, moral sensitivity, favorable views on feminism and the women’s movement, and a propensity to participate in feminist activities, are presumed to positively correlate with their perceptions of sexual harassment and their allocation of blame or sympathy toward the victim. Conversely, other characteristics, such as belief in sexual harassment myths, sexist attitudes, and sexual narcissism, are expected to negatively influence these perceptions and attributions.

## Materials and methods

### Study design and participants

**FIGURE 1:**
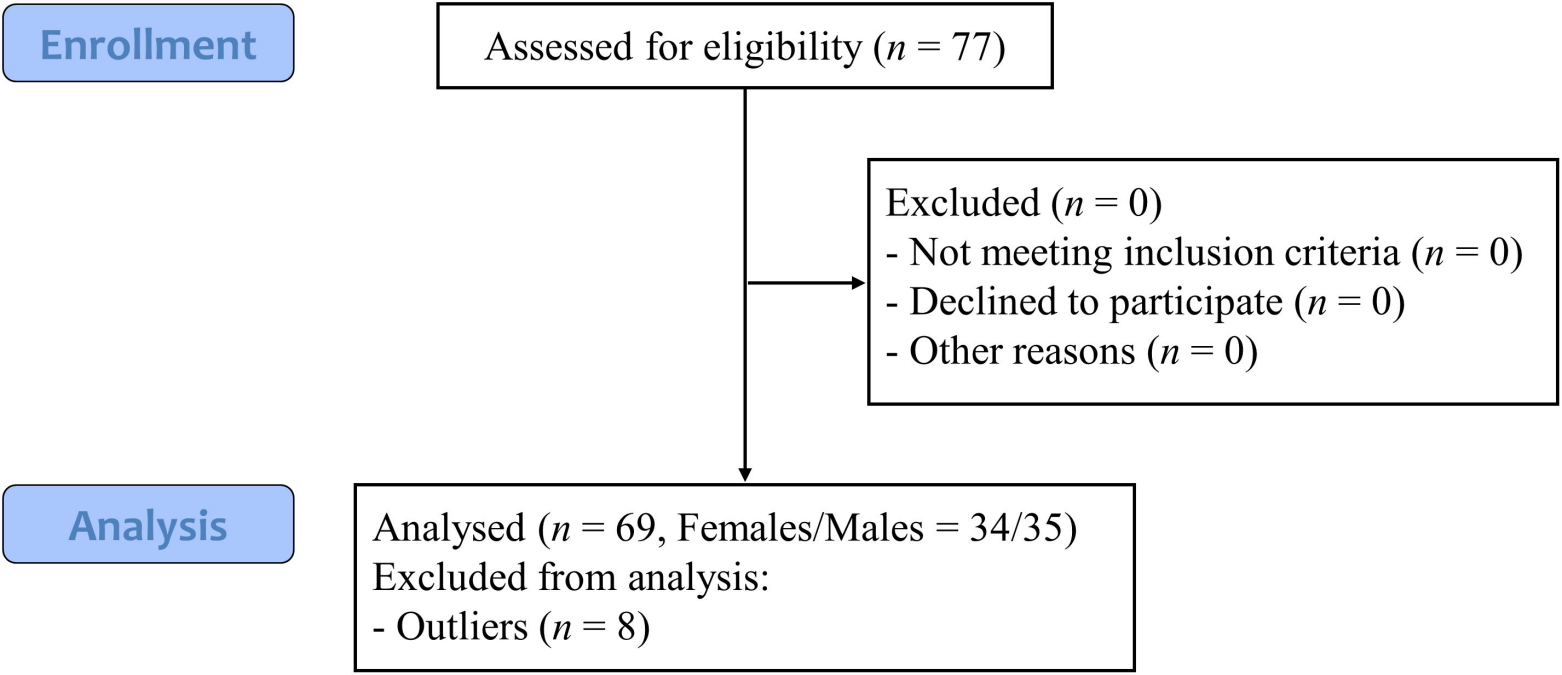
Participant sampling flow diagram.

This study employed a mixed experimental design with both within-subjects (sexual harassment type: gender harassment, unwanted sexual attention, sexual coercion) and between-subjects (observer sex: male, female) factors. The repeated-measures design enhances statistical power for detecting within-subjects factor effects (harassment type) relative to between-subjects designs, while simultaneously yielding multiple observations per participant. A sample size estimation using G*Power (*F*-tests, ANOVA: Repeated measures, within-between interaction) indicated that 44 participants were required to achieve 95% power for detecting moderate effects (*f* = 0.25, α = 0.05). To account for potential dropouts and maintain the sample size above this threshold, 77 healthy, right-handed Chinese university students (mean age = 20.40 ± 2.10 years; 38 males) were enrolled. Participants underwent careful screening and completed validated scales to ensure they were within normal ranges for anxiety, depression, and mood, minimizing confounding effects. They completed the State-Trait Anxiety Inventory (STAI; Spielberg et al., 1983), the Beck’s Depression Inventory (BDI; Beck et al., 1996) and the Positive and Negative Affect Schedule (PANAS; Watson et al., 1988). All participants scored within normal ranges on these measures, confirming the absence of psychiatric conditions (Beck et al., 1996; Baron-Cohen et al., 2001). 8 participants identified as extreme outliers (values exceeding Q3 + 3 × IQR or below Q1−3 × IQR) were excluded from the analysis, leading to a final sample size of *N* = 69 (35 males). Detailed inclusion and exclusion criteria are presented in Figure 1. Ethical approval was obtained from the Medical Ethics Committee of Shenzhen University (No: PN-202200109) and were in accordance with the latest revision of the Declaration of Helsinki. Written informed consent emphasized voluntary participation and unrestricted withdrawal, and was obtained from all participants prior to their inclusion in the study.

### Experimental procedures

Participants completed the experiment individually in a controlled laboratory setting (mean duration: 90 min). Each participant experienced 85 sexual harassment scenarios from the first-person perspective of the female survivors. Scenarios were categorized into three types: gender harassment (*n* = 31), unwanted sexual attention (*n* = 27), and sexual coercion (*n* = 27). These scenarios were derived from real incidents reported by females online, reflecting the typical pattern of male-to-female sexual harassment. All scenarios followed a standardized format specifying: (a) Context (time/location), (b) Harasser actions, (c) Victim outcomes, and (d) Victim emotional responses. A sample item from the sexual coercion scenarios is “As an actress, I encountered a distressing incident one evening. The director, under the guise of kindness, offered to drive my colleagues and me home. Residing furthest away, I was last to be dropped off. Unexpectedly, he detoured to a secluded area and climbed into the back seat, where he subjected me to unwanted advances, promising prominent roles in exchange for sexual compliance. Overcome with fear, I protested, citing a boyfriend who awaited my return. The director, visibly displeased, eventually conceded and drove me home. Alone, I sought refuge in the bathroom, succumbing to tears and consumed by trepidation. The incident left me too intimidated to confide in my boyfriend. Resolute, I terminated my association with the production the following day.” After reading each scenario, the participants rated it on four aspects:

#### Emotional reactions

Participants first rated their emotional responses along two dimensions: valence (pleasure-displeasure continuum) and arousal (activation intensity), using separate 9-point Likert scales for each dimension. Emotional valence describes the extent to which an emotion is positive or negative, whereas arousal measures how intense or stimulating an emotion is, or how much it activates the body and mind (Lang et al., 1997). The valence scale ranged from 1 (very negative) to 9 (very positive), with 5 indicating a neutral emotion. The arousal scale ranged from 1 (calm) to 9 (highly aroused).

#### Perception of sexual harassment

Participants were asked to indicate the extent to which they perceived the scenario as sexual harassment, using a 9-point Likert scale from 1 (definitely is not sexual harassment) to 9 (definitely is sexual harassment).

#### Victim blaming

Participants were asked to indicate how much responsibility they assigned to the victim in the scenario, using a 9-point Likert scale from 1 (no responsibility) to 9 (full responsibility).

#### Victim sympathy

Participants were asked to indicate how much sympathy they felt for the victim in the scenario, using a 9-point Likert scale from 1 (no sympathy) to 9 (full sympathy).

### Psychometric assessment

#### Empathy

The Interpersonal Reactivity Index (IRI) was administered to assess empathy (Siu and Shek, 2005). This 22-item self-report instrument comprises four subscales: Fantasy, Perspective Taking, Empathetic Concern, and Personal Distress. The Chinese version of the IRI was developed by Siu and Shek (2005), and its psychometric properties have been empirically substantiated. The Cronbach’s alpha coefficient for this scale in the present study was 0.794.

#### Attitudes toward feminism and the women’s movement

The attitudes toward feminism and the women’s movement (FWM) scale was administered to measure participants’ affective attitudes toward the feminist movement (Fassinger, 1994). This scale consists of 10 items, of which 6 are statements that favor feminism and 4 are statements that oppose feminism. The participants need to choose their attitudes on a 5-point scale. The Chinese version of the FWM was developed and validated by Chen and Zheng (2023), with empirical evidence supporting its psychometric properties. The Cronbach’s alpha coefficient for this scale in the present study was 0.683.

#### Willingness to engage in feminist behaviors

The WEFB scale was administered to measure participants’ willingness to engage in feminist behaviors (Redford et al., 2018). This scale consists of 11 items such as “How willing would you be to bring up feminist issues in conversation with someone you know well?” and “Imagine a feminist organization has contacted you to ask you to add your name to their membership list. How willing would you be to do so?”. The Chinese version of the WEFB was developed and validated by Liu and Zheng (2019), with empirical evidence supporting its psychometric properties. The Cronbach’s alpha coefficient for this scale in the present study was 0.921.

#### Moral sensitivity

The Dispositional Moral Sensitivity Questionnaire (DMSQ) was administered to measure a tendency toward reflection and the ability to detect and explain moral problems (Zheng and Cen, 2008). The DMSQ is a 28-item self-reported questionnaire that encompasses five factors: Empathic Guilt, Punishment, Intrusiveness of Empathy, Frequency of Perception, and Sympathetic Imagination. A higher total score indicates a greater disposition toward awareness and understanding of moral issues. Zheng and Cen (2008) developed and validated the DMSQ, establishing its psychometric properties through empirical validation. The Cronbach’s alpha coefficient for this scale in the present study was 0.902.

#### Sexism

The Ambivalent Sexism Inventory (ASI) was administered to assess hostile (overtly negative attitudes) and benevolent sexism (attitudes seem subjectively positive but are actually harmful) toward women (Glick and Fiske, 1996). The ASI consists of 22 items divided into two subscales: Hostile Sexism, which reflects beliefs in traditional gender roles, the perception of women as inferior, and the view that women manipulate men through their sexuality; and Benevolent Sexism, which reflects protective and chivalrous attitudes, the idealization of women as pure and nurturing, and the belief that women should be taken care of and provided for by men. The Chinese version of the ASI was developed and validated by Chen et al. (2009), with empirical evidence supporting its psychometric properties. The Cronbach’s alpha coefficient for this scale in the present study are 0.859.

#### Sexual harassment myth

The Illinois Sexual Harassment Myth Acceptance (SHMA) Scale was administered to measure the acceptance of sexual harassment mythology (Lonsway et al., 2008). The SHMA contained 20 myth items, tapping into beliefs about Fabrication/Exaggeration (eight items), Ulterior Motives (five items), Natural Heterosexuality (four items), Woman’s Responsibility (three items), and four negatively worded filler items to reduce response bias. The Chinese version of the SHMA was developed and validated by Yang (2021), with empirical evidence supporting its psychometric properties, and has been successfully used with Chinese respondents (Zhang et al., 2025). The Cronbach’s alpha coefficient for this scale in the present study was 0.942.

#### Sexual harassment attitude

The Sexual Harassment Attitude Scale (SHAS) consisting of 19 items and measured participants’ tolerance of sexual harassment (Mazer and Percival, 1989). High scores on the SHAS indicate more acceptance and tolerance of sexual harassment and less agreement with contemporary feminist understandings of its causes. The Chinese version of the SHAS was developed and validated by Shi and Zheng (2020), with empirical evidence supporting its psychometric properties. The Cronbach’s alpha coefficient for this scale in the present study was 0.842.

#### Sexual narcissism

The Sexual Narcissism Scale (SNS) was administered to measure narcissism in the sexual domain (Widman and McNulty, 2010). SNS is a 20-item self-reported questionnaire that encompasses four subscales: Sexual Exploitation, Sexual Entitlement, Low Sexual Empathy, and Grandiose Sense of Sexual Skill. The Chinese version of the SNS was developed and validated by Liu and Zheng (2020), with empirical evidence supporting its psychometric properties. The Cronbach’s alpha coefficient for this scale in the present study was 0.703.

### Data analysis

Separate 2 × 3 repeated-measures analyses of variance (ANOVAs) were performed for each dependent variable (valence, arousal, perception of harassment, victim sympathy, and victim blaming) to assess interactions between observers’ sex (between-subjects factor: male, female) and sexual harassment type (within-subjects factor: gender harassment, unwanted sexual attention, sexual coercion). Significant effects of observers’ sex were further examined using *post hoc* independent-samples *t*-tests (two-tailed). All analyses were conducted in SPSS 27.0 (IBM Corp., Armonk, NY, United States).

Prior to conducting ANOVA, the normality assumption was evaluated using Kolmogorov-Smirnov tests. For variables satisfying normality, sphericity was assessed via Mauchly’s test. When sphericity violations were detected, multivariate analysis of variance (MANOVA) was employed as it does not require the sphericity assumption. To address potential bias from non-normality, generalized estimating equations (GEE) were subsequently implemented for sensitivity analysis. The GEE results corroborated the primary ANOVA/MANOVA findings (Supplementary Table 1), demonstrating robustness against violations of distributional assumptions.

## Results

### Demographics and questionnaires

The males (*n* = 35) and females (*n* = 34) participants did not differ significantly in age, levels of depression, mood and anxiety (see Table 1 for detailed group characteristics). However, they differed significantly in psychological and attitudinal factors. Female participants showed higher empathy ability (*P* = 0.024), more positive attitudes toward feminism (*P* < 0.001), and more WEFB (*P* < 0.001). Male participants showed higher sexism (*P* < 0.001), tolerance of sexual harassment (*P* < 0.001), sexual narcissism (*P* = 0.035), and sexual harassment myth acceptance (*P* < 0.001).

**TABLE 1:** Demographics and questionnaires.

Variables	Females (*n* = 34)	Males (*n* = 35)	*T*	*P*
Age (years)	19.97 ± 1.83	20.74 ± 2.25	−1.559	0.124
STAI-state	35.18 ± 9.85	33.97 ± 8.86	0.534	0.595
STAI-trait	39.56 ± 10.77	37.00 ± 8.50	1.097	0.277
BDI-II	6.29 ± 7.53	5.03 ± 6.39	0.753	0.454
PANAS-positive	28.47 ± 6.83	29.14 ± 7.11	−0.400	0.690
PANAS-negative	13.62 ± 4.78	14.69 ± 5.70	−0.842	0.403
IRI	56.00 ± 11.77	50.37 ± 8.25	2.305	0.024
DMSQ	13.28 ± 3.37	13.08 ± 3.18	0.251	0.803
FWM	37.09 ± 4.20	32.60 ± 3.12	5.054	<0.001
WEFB	50.24 ± 8.77	38.83 ± 7.84	5.701	<0.001
ASI	34.26 ± 11.36	53.09 ± 11.37	−6.877	<0.001
SHMA	34.88 ± 10.60	48.37 ± 11.40	−5.087	<0.001
SNS	35.38 ± 7.59	39.17 ± 7.05	−2.149	0.035
SHAS	40.18 ± 6.75	49.49 ± 10.34	−4.415	<0.001

STAI, State-Trait Anxiety Inventory; BDI-II, Beck’s Depression Inventory-II; PANAS, Positive and Negative Affect Schedule; IRI, Chinese version of the Interpersonal Reactivity Index; DMSQ, Dispositional Moral Sensitivity Questionnaires; FWM, attitudes toward feminism and the women’s movement; WEFB, willingness to engage in feminist behaviors; ASI, Ambivalent Sexism Inventory; SHAS, The Sexual Harassment Attitude Scale; SNS, Sexual Narcissism Scale; SHMA, The Illinois Sexual Harassment Myth Acceptance Scale. Data are expressed as mean ± SD (SD: standard deviation). For continuous variables, independent sample *t*-tests were carried out.

### Gender differences in emotional reactions, perception of sexual harassment, victim sympathy, and victim blaming

#### Emotional reactions

**FIGURE 2:**
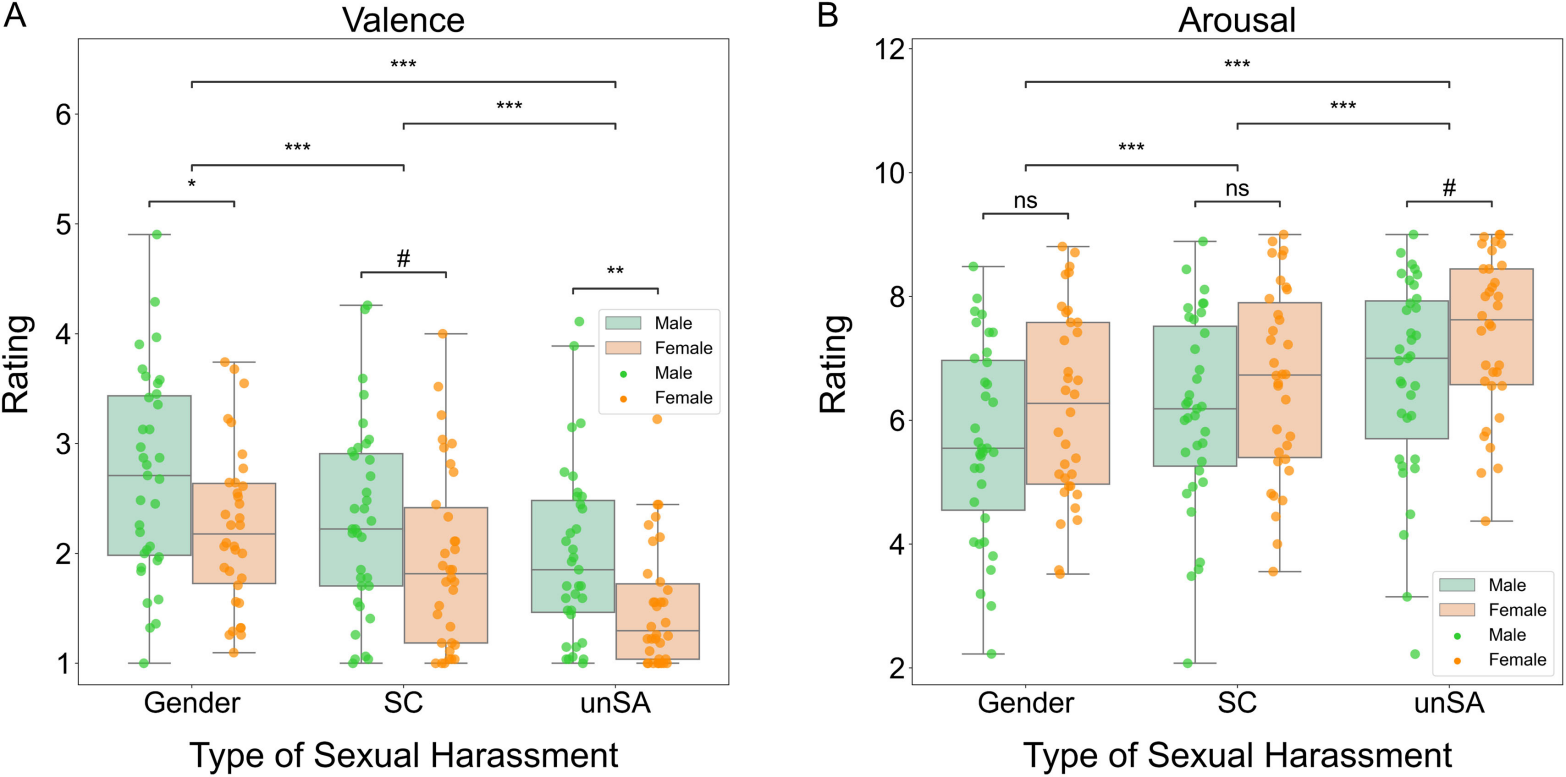
Box plots for significant differences in emotional reactions. **(A)** Valence ratings. **(B)** Arousal ratings. Gender, gender harassment; SC, sexual coercion; unSA, unwanted sexual attention. ns, non-significant, ^#^*P* < 0.1, *P < 0.05, **P < 0.01, ***P < 0.001.

We examined the valence ratings using a two-way repeated measures ANOVA and found significant main effects of sexual harassment type (*F*[2, 66] = 121.016, *P* < 0.001, η^2^_P_ = 0.644) and observers’ sex (*F*[1, 67] = 5.914, *P* = 0.018, η^2^_P_ = 0.081). However, the interaction between these two factors was not significant (*F*[2, 66] = 0.912, *P* = 0.404, η^2^_P_ = 0.013). *Post-hoc* tests showed that the three types of sexual harassment scenarios differed significantly in their valence ratings. Specifically, the unwanted sexual attention scenarios were rated as the most negative, followed by the sexual coercion scenarios and then the gender harassment scenarios (all *P*s < 0.001). Moreover, female participants rated the unwanted sexual attention and gender harassment scenarios more negatively than male participants (*P* = 0.005 and *P* = 0.020, respectively), but there was marginally significant gender difference in the ratings for the sexual coercion scenarios (*P* = 0.071). Figure 2A summarizes these results schematically.

Similarly, we analyzed the arousal ratings using a two-way repeated measures ANOVA. We found a significant main effect of sexual harassment type (*F*[2, 66] = 55.396, *p* < 0.001, η^2^_P_ = 0.627). However, neither the main effect of observers’ sex (*F*[1, 67] = 2.794, *P* = 0.099, η^2^_P_ = 0.040) nor the interaction between these two factors (*F*[2, 66] = 1.156, *P* = 0.321, η^2^_P_ = 0.034) was significant. *Post-hoc* tests showed that the three types of sexual harassment scenarios differed significantly in their arousal ratings. Specifically, the unwanted sexual attention scenarios were rated as the most arousing, followed by the sexual coercion scenarios and then the gender harassment scenarios (all *P*s < 0.001). Figure 2B summarizes these results schematically.

#### Perception of sexual harassment

**FIGURE 3:**
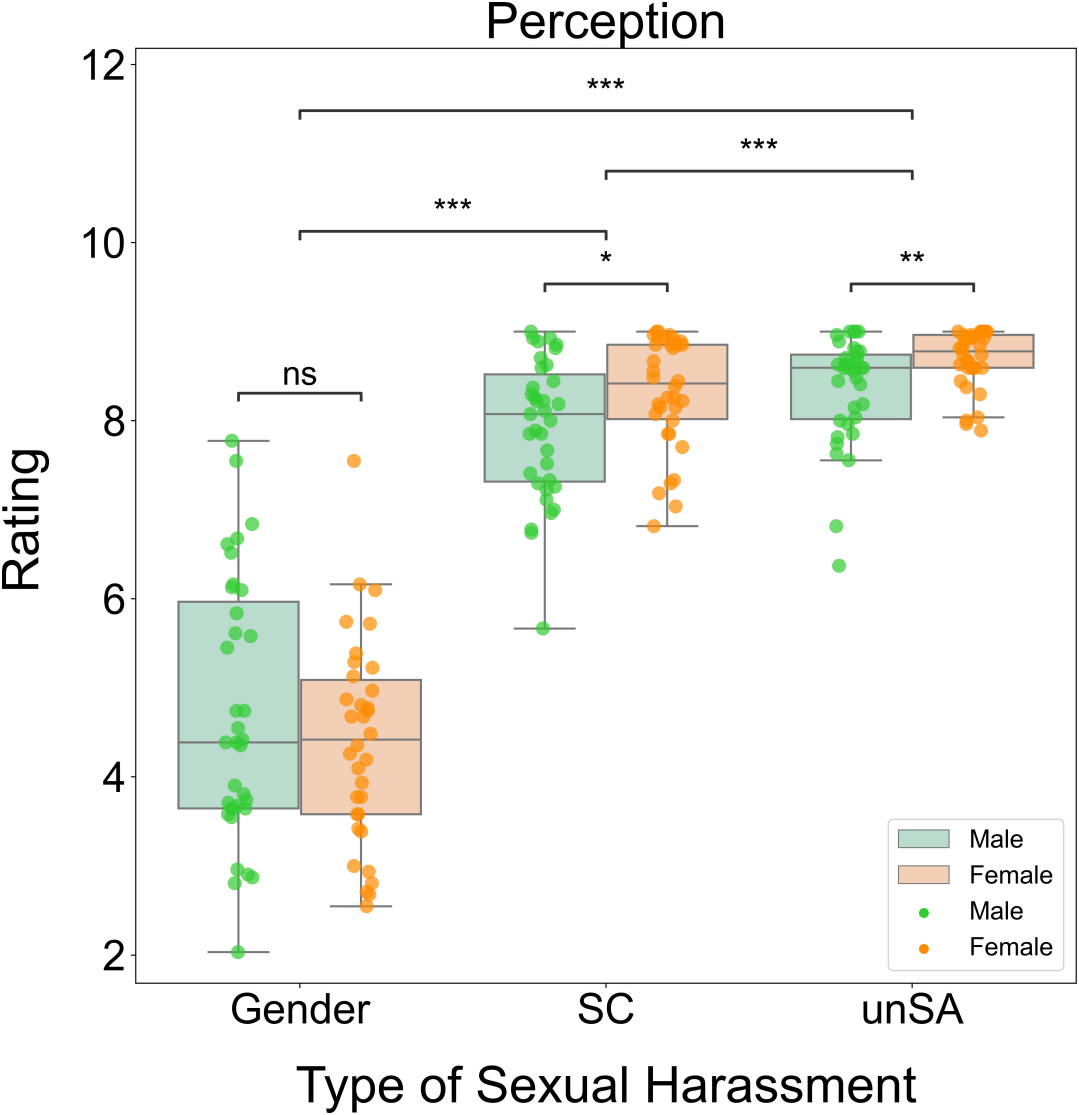
Box plots for significant differences in perception of sexual harassment. Gender, gender harassment; SC, sexual coercion; unSA, unwanted sexual attention. ns, non-significant, *P < 0.05, **P < 0.01, ***P < 0.001.

We performed a two-way repeated measures ANOVA to investigate how sexual harassment type and observers’ sex influenced perception of harassment. We found a significant main effect of sexual harassment type (*F*[2, 66] = 329.553, *P* < 0.001, η^2^_P_ = 0.909). However, the main effect of observers’ sex was not significant (*F*[1, 67] = 0.737, *P* = 0.394, η^2^_P_ = 0.011). The interaction effect between these two factors was marginally significant (*F*[2, 66] = 2.707, *P* = 0.074, η^2^_P_ = 0.076). Further simple effects analysis showed that female observers perceived sexual coercion (*P* = 0.023) and unwanted attention (*P* = 0.007) as more severe forms of sexual harassment than male observers did, whereas there was no significant gender difference in the perception of gender harassment (*P* = 0.321). This indicates that female observers were more sensitive to sexual coercion and unwanted attention than male observers. Additionally, the perception ratings differed significantly across the three types of sexual harassment scenarios. Specifically, the unwanted sexual attention scenarios were perceived as the most severe forms of sexual harassment, followed by the sexual coercion scenarios and then the gender harassment scenarios (all *P*s < 0.001). Figure 3 summarizes these results schematically.

#### Victim sympathy

**FIGURE 4:**
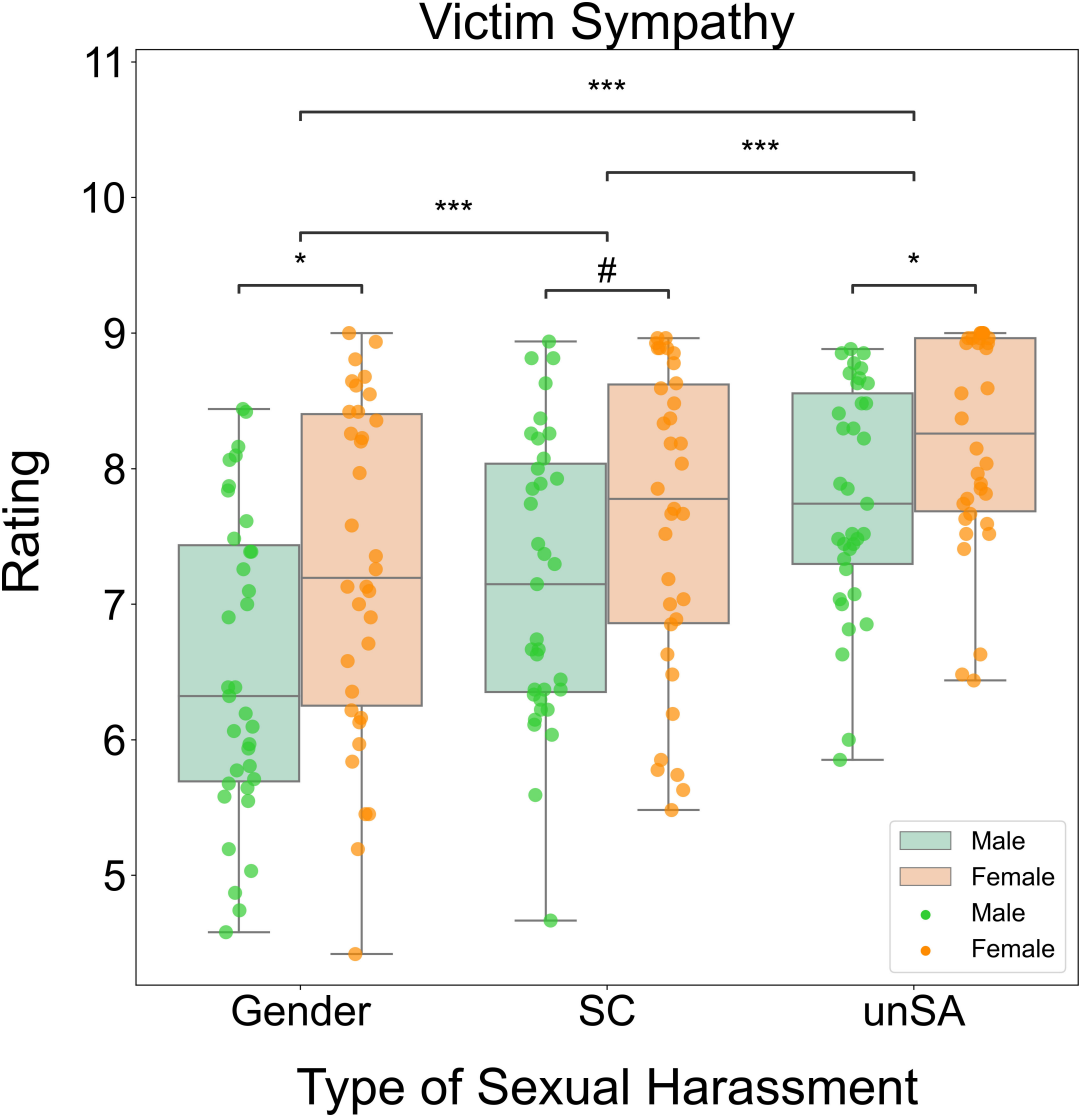
Box plots for significant differences in victim sympathy. Gender, gender harassment; SC, sexual coercion; unSA, unwanted sexual attention. ^#^*P* < 0.1, *P < 0.05, ***P < 0.001.

We conducted a two-way repeated measures ANOVA to investigate how sexual harassment type and observers’ sex influenced victim sympathy. We found significant main effects of sexual harassment type (*F*[2, 66] = 59.419, *P* < 0.001, η^2^_P_ = 0.643) and observers’ sex (*F*[1, 67] = 5.278, *P* = 0.025, η^2^_P_ = 0.073). However, the interaction effect between these two factors was not significant (*F*[2, 66] = 2.394, *P* = 0.099, η^2^_P_ = 0.068). *Post-hoc* tests showed that the unwanted sexual attention scenarios elicited the highest victim sympathy ratings, followed by the sexual coercion scenarios and then the gender harassment scenarios (all *P*s < 0.001). Additionally, female observers expressed higher victim sympathy ratings than male observers for the gender harassment (*P* = 0.012) and unwanted attention (*P* = 0.034), but there was marginally significant gender difference for the sexual coercion scenarios (*P* = 0.092). Figure 4 summarizes these results schematically.

#### Victim blaming

**FIGURE 5:**
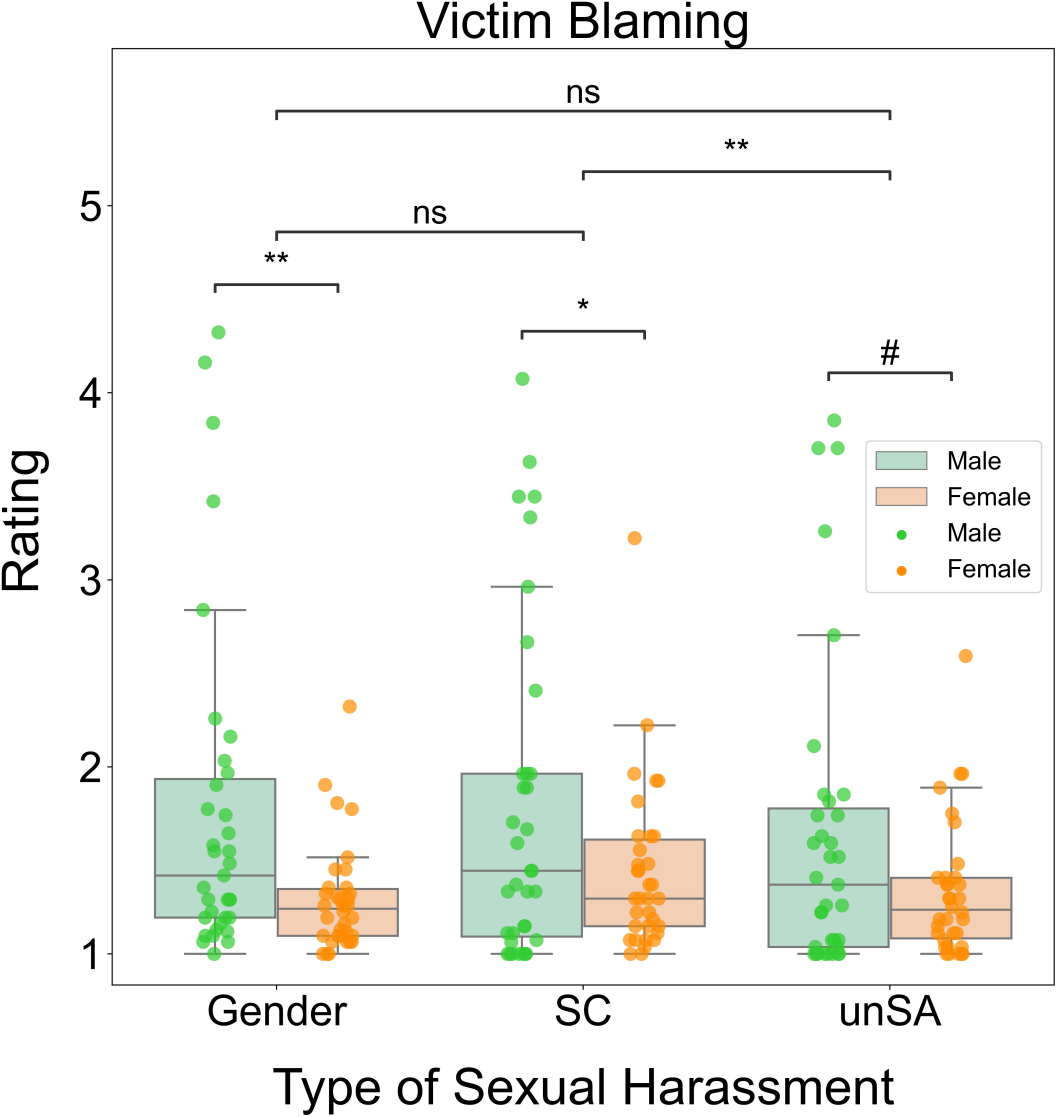
Box plots for significant differences in victim blaming. Gender, gender harassment; SC, sexual coercion; unSA, unwanted sexual attention. ns, non-significant, ^#^*P* < 0.1, *P < 0.05, **P < 0.01.

We performed a two-way repeated measures ANOVA to investigate how sexual harassment type and observers’ sex influenced victim blaming. We found significant main effects of sexual harassment type (*F*[2, 66] = 5.578, *P* = 0.005, η^2^_P_ = 0.077) and observers’ sex (*F*[1, 67] = 5.778, *P* = 0.019, η^2^_P_ = 0.079). However, the interaction effect between these two factors was not significant (*F*[2, 66] = 2.484, *P* = 0.087, η^2^_P_ = 0.036). *Post-hoc* tests indicated that sexual coercion elicited significantly higher victim blaming than unwanted sexual attention (*P* = 0.001). There was no significant difference in victim blaming between sexual coercion and gender harassment (*P* = 0.154), or between gender harassment and unwanted sexual attention (*P* = 0.826). Moreover, male observers blamed the victims more than female observers for all three types of sexual harassment. The gender differences in victim blaming were significant for gender harassment (*P* = 0.005) and sexual coercion (*P* = 0.045), and marginally significant for unwanted sexual attention (*P* = 0.053). Figure 5 shows a schematic summary of these results.

### Factors affecting emotional reactions, perception of sexual harassment, victim sympathy, and victim blaming

**FIGURE 6:**
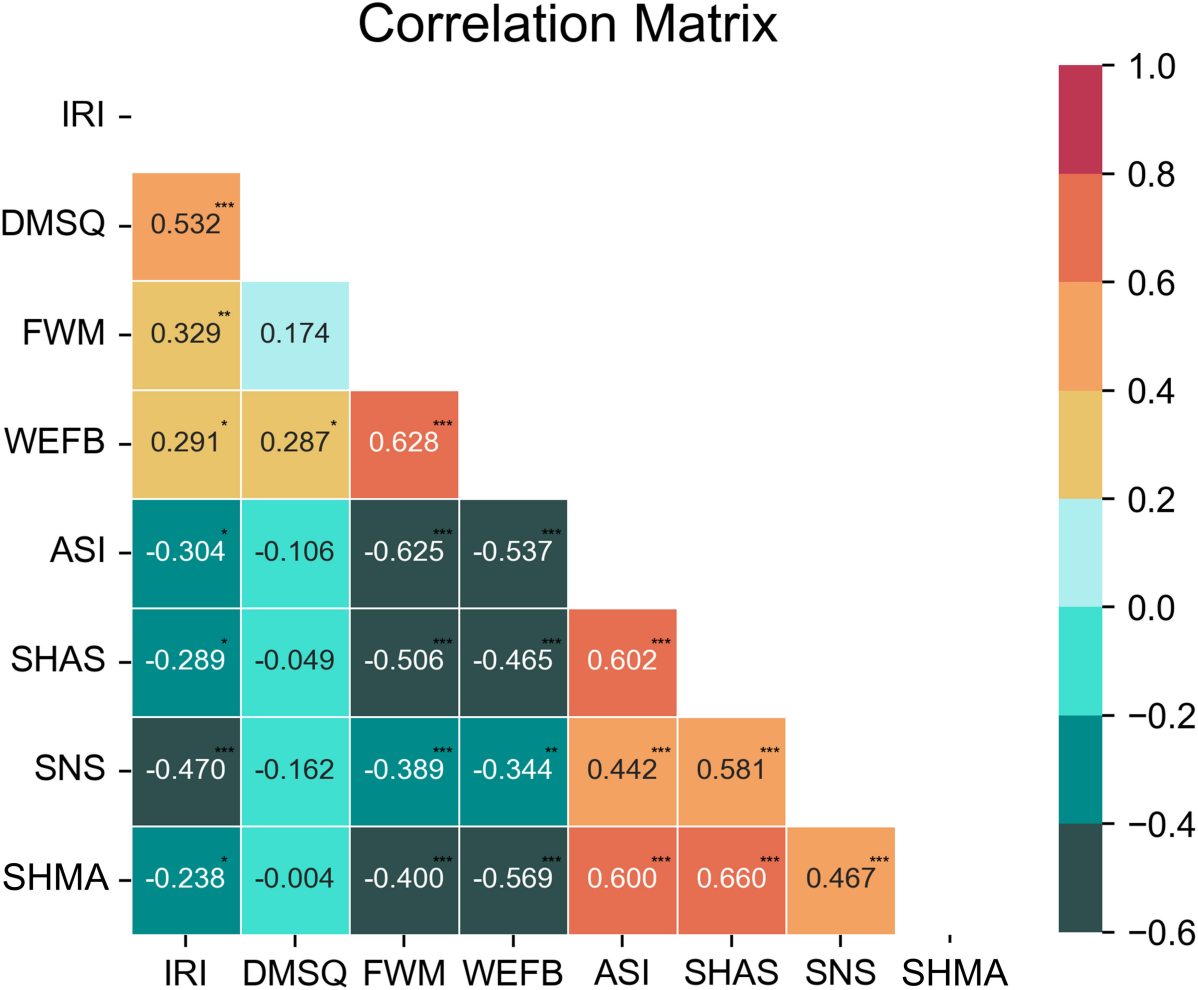
Correlations among the main scales. IRI, Interpersonal Reactivity Index; DMSQ, Dispositional Moral Sensitivity Questionnaires; FWM, Attitudes Toward Feminism and the Women’s Movement; WEFB, Willingness to Engage in Feminist Behaviors; ASI, Ambivalent Sexism Inventory; SHAS, Sexual Harassment Attitude Scale; SNS, Sexual Narcissism Scale; SHMA, Illinois Sexual Harassment Myth Acceptance Scale. *P < 0.05, **P < 0.01, ***P < 0.001.

**FIGURE 7:**
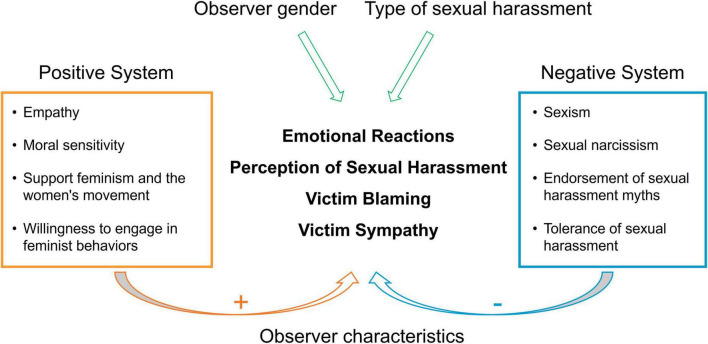
A schematic overview of the two divergent belief and value systems that influence observers’ perceptions and responses to sexual harassment.

This study examined the influence of psychological and attitudinal factors on the perception and response to different sexual harassment scenarios. The results presented above showed that gender harassment was less likely to be perceived as sexual harassment than other forms of harassment. Thus, we distinguished gender harassment from unwanted sexual attention and sexual coercion. We computed two measures of perception: one based on the average of all three forms of harassment (perception1), and one based on the average of only unwanted sexual attention and sexual coercion (perception2). Consistent with our hypotheses, we found that higher empathy ability, moral sensitivity, positive attitudes toward feminism and the women’s movement, and feminist engagement were associated with more accurate perception of sexual harassment, higher sympathy for the victim, and lower victim blaming. Conversely, higher sexism, sexual narcissism, tolerance of sexual harassment, and acceptance of sexual harassment myths were associated with less accurate perception of sexual harassment, lower sympathy for the victim, and higher victim blaming. These findings suggest that individual differences in psychological and attitudinal factors affect how people perceive and respond to sexual harassment incidents. Table 2 displays the correlations among all the main variables in this study. Figure 6 illustrates the correlations among the main scales used in this study. Figure 7 presents a schematic depiction of two opposing belief and value systems shaping observers’ perceptions and reactions: a positive system, characterized by respect and concern for the rights and dignity of the victims, and a negative system, marked by disregard and indifference to those same rights and dignity.

**TABLE 2:** Correlations among measures.

Variables	Valence	Arousal	Perception1	Perception2	Victim sympathy	Victim blaming
IRI	−0.380***	0.413***	0.204	0.394***	0.438***	−0.024
DMSQ	−0.295*	0.357**	0.226	0.279*	0.407***	−0.008
FWM	−0.300*	0.280*	0.171	0.378**	0.312**	−0.278*
WEFB	−0.384**	0.372**	0.371**	0.407***	0.437***	−0.235
ASI	0.395***	−0.376**	−0.211	−0.516***	−0.504***	0.372**
SHAS	0.357**	−0.365**	−0.236	−0.551***	−0.389***	0.449***
SNS	0.369**	−0.385**	−0.259*	−0.510***	−0.417***	0.318**
SHMA	0.478***	−0.393***	−0.238*	−0.481***	−0.479***	0.338**

IRI, Interpersonal Reactivity Index-C; DMSQ, Dispositional Moral Sensitivity Questionnaires; FWM, attitudes toward feminism and the women’s movement; WEFB, willingness to engage in feminist behaviors; ASI, Ambivalent Sexism Inventory; SHAS, The Sexual Harassment Attitude Scale; SNS, Sexual Narcissism Scale; SHMA, The Illinois Sexual Harassment Myth Acceptance Scale.

*P < 0.05,

**P < 0.01,

***P < 0.001.

## Discussion

The variance in observers’ perceptions and reactions to sexual harassment, notably in terms of victim sympathy and victim blaming, has been extensively documented. However, the underlying mechanisms that drive these differences remain elusive. This study delves into the potential factors that may account for these disparities, drawing on a cohort of Chinese college students. The findings are multifaceted: (1) Gender harassment is generally perceived as less severe than unwanted sexual attention and sexual coercion; (2) Male observers are more likely to understate the severity of sexual harassment and assign greater blame to victims compared to female observers; (3) Female observers experience more intense emotional reactions to sexual harassment and demonstrate increased sympathy for victims; (4) Female participants exhibit higher levels of empathy ability, favorable attitudes toward feminism, and a readiness to participate in feminist actions, while male participants show greater tolerance for sexual harassment, endorsement of sexual harassment myths, sexist beliefs, and sexual narcissism; (5) Attributes such as empathy, moral sensitivity, and supportive attitudes toward feminism positively influence perceptions and attitudes toward sexual harassment; (6) Conversely, tolerance for sexual harassment, belief in sexual harassment myths, sexism, and sexual narcissism contribute to more negative perceptions and attitudes. These insights offer valuable guidance for crafting effective interventions to prevent and address sexual harassment.

### Factors affecting observers’ perception of sexual harassment, emotional reactions, victim blaming, and victim sympathy

This study examined the influence of different types of sexual harassment on observers’ perceptions of the harassment, emotional responses, and tendencies toward victim blaming and sympathy. In line with previous studies (Saunders and Senn, 2009; Herrera Enríquez et al., 2018), gender harassment was deemed less severe than unwanted sexual attention, likely due to its non-sexual motivations. Diverging from earlier studies, this study also considered sexual coercion as a separate category of sexual harassment. Findings indicate that unwanted sexual attention is perceived as the most severe form of harassment, followed by sexual coercion, and then gender harassment. This study incorporated a minimum of 27 scenarios for each facet of sexual harassment, with a broad spectrum of severity observed. Instances of sexual coercion ranged from verbal bribes and threats to those entailing physical sexual contact. Predominantly, scenarios involving unwanted sexual attention included invasive physical contact, thereby constituting the most egregious level of sexual harassment.

While previous studies (Saunders and Senn, 2009; Herrera Enríquez et al., 2018) focused on the perceptions of observers from a single gender without exploring differences between genders, this study corroborates the documented gender differences in harassment perception. Women generally identifying a wider spectrum of behaviors as harassment (Rotundo et al., 2001; Rothgerber et al., 2021; Hehman et al., 2022), whereas men tended to minimize the seriousness of harassment incidents. Studies examining gender differences across the type of harassment have yielded inconsistent results (Rotundo et al., 2001; Rothgerber et al., 2021). A meta-analysis by Rotundo et al. (2001) revealed that gender difference was larger for less extreme and more ambiguous behaviors, such as derogatory attitudes and dating pressure, than for sexual propositions and sexual coercion. Conversely, Rothgerber et al. (2021) observed smaller gender differences in perceptions of less severe forms of sexual harassment, such as derogatory attitudes and non-sexual physical contact. Given the temporal gap since Rotundo et al.’s (2001) study, their findings warrant cautious interpretation due to potential shifts in social norms. Rothgerber et al. (2021) also analyzed the gender differences in terms of three dimensions of sexual harassment (gender harassment vs. unwanted sexual attention vs. sexual coercion). However, they did not find a significant interaction between participant gender and sexual harassment dimension. Our study diverges from Rothgerber et al. (2021) by identifying a pronounced gender difference in the perceptions of unwanted sexual attention, with less disparity noted in gender harassment cases. This suggests that gender differences amplify as behaviors are more clearly defined as sexual harassment. Possible explanations for these discrepancies include the scope of harassment cases examined. Rothgerber et al. (2021) limited their analysis to a narrow set of sexual harassment instances (one scenario of sexual coercion and two scenarios of gender harassment), whereas our study encompassed a broader range with varying severity. Additionally, we incorporated victims’ emotional responses post-scenario to enhance realism, which may influence observers’ perceptions and reactions to harassment cases.

This study investigated the impact of various psychological and attitudinal factors on observers’ perceptions of sexual harassment, emotional reactions, sympathy for the victim, and blame attribution. The results revealed that observers characterized by heightened empathy ability and moral sensibility, supportive attitudes toward feminism, and a greater propensity to engage in feminist behaviors tended to perceive sexual harassment with increased sensitivity, exhibited stronger emotional reactions, extend greater sympathy toward the victim, and were less likely to attribute blame to the victim. These attributes are positively interrelated, establishing a system that mirrored respect and concern for the victim’s rights and dignity, as well as awareness of the harm and injustice of sexual harassment. Conversely, observers characterized by heightened sexism, sexual narcissism, belief in sexual harassment myths, and tolerance of sexual harassment perceived sexual harassment less sensitively, diminished sympathy for the victim, and a greater propensity to blame the victim. These factors were also interconnected, forming a detrimental system that reflected disregard and indifference for the victim’s rights and dignity, as well as denial or rationalization of the harm and injustice of sexual harassment.

The study further explored the relationship between the two aforementioned systems, revealing a negative correlation that suggests they have contrasting effects on perceptions of sexual harassment. Consistent with previous studies, our results suggest that a lower endorsement of rape/sexual harassment myths correlates with heightened empathy ability (Diehl et al., 2014; Long and Herr, 2022), whereas a greater acceptance of these myths is associated with increased sexism (Lonsway et al., 2008) and sexual narcissism (Long and Herr, 2022). Furthermore, a higher tolerance for sexual harassment aligns with a stronger belief in rape/sexual harassment myths (LeMaire et al., 2016). This study enriches the sexual harassment discourse by introducing moral sensitivity, positive attitudes toward feminism, and a propensity for feminist engagement as influential psychological and attitudinal factors in observers’ perceptions of sexual harassment. Observers who are supportive of feminism and inclined to participate in feminist activities tend to exhibit more robust gender equality stances, reduced sexism, and enhanced sympathy toward female victims. Similarly, those with greater moral sensitivity also demonstrate increased sympathy toward female victims. The study identified significant gender differences in these psychological and attitudinal factors. Female participants exhibited higher scores in the positive system, encompassing empathy ability, favorable attitudes toward feminism, and willingness to engage in feminist behavior. Conversely, male participants scored higher in the negative system, characterized by sexism, tolerance of sexual harassment, sexual narcissism, and sexual harassment myth acceptance. These psychological and attitudinal factors may account for the observed gender differences in the perception of sexual harassment, emotional reactions, attribution of blame, and sympathy toward the victim.

### Intervention strategies for sexual harassment

Although substantial research exists on sexual harassment intervention strategies in Western contexts (Mujal et al., 2021; Jenner et al., 2022), related studies in China remain scarce (Chen et al., 2022). Moreover, current intervention strategies demonstrate limited awareness among Chinese university students (He et al., 2024). Implementing preventive interventions to improve observers’ perceptions and attitudes toward sexual harassment constitutes a core approach to prevent sexual harassment. Our findings underscore the necessity for targeted interventions addressing foundational belief and value systems that shape observers’ perception and response to sexual harassment. Drawing on our dual-systems framework and empirical evidence, we propose the following interventions: (a) Embed gender equality curricula coupled with moral sensitivity modules in university core requirements, targeting the deconstruction of regressive gender schemas through critical reflection exercises; (b) Implement mandatory bystander-empowerment programs featuring evidence-based myth deconstruction, particularly in high-tolerance male-dominated fields; (c) Develop culturally adapted interventions that reframe harassment prevention as collective integrity protection (e.g., “social responsibility narratives” in Confucian contexts); (d) Institutionalize perspective-taking simulations using virtual reality scenarios to enhance empathy and intervention self-efficacy campus-wide; (e) Conduct cognitive restructuring workshops addressing sexual narcissism and hostile sexism, utilizing behavioral commitment techniques to sustain attitude change.

## Limitations

Several potential limitations should be noted. The modest sample size of exclusively young Chinese university students constrains broad generalizability and sophisticated moderation/mediation analyses, though our powerful repeated-measures design ensured robust detection of key effects through high-density within-participant observations. Demographic representativeness is further limited given documented cultural variations in sexual harassment perceptions, empathy processing, and victim blaming (Kennedy and Gorzalka, 2002; Van der Bruggen and Grubb, 2014; Atkins et al., 2016; Shi and Zheng, 2021), compounded by participants’ distinct attitudinal profile—characterized by higher education-associated liberalism, reduced stereotype acceptance, and heightened victim supportiveness. Future investigations should recruit larger, demographically diverse samples to address these constraints. Despite these limitations, this study establishes an empirically grounded foundation for understanding sexual harassment perceptions among Chinese university youth through its methodologically rigorous approach incorporating GEE validation, theoretically meaningful effect sizes for core hypotheses, and culturally significant contributions within an under-researched context–providing critical insights into a demographic actively engaged in social transformation. Additionally, the study may be subject to social desirability biases, which could distort the results (Larson, 2019). Although confidentiality assurances were implemented alongside honesty priming procedures to mitigate social desirability bias, self-reported questionnaire data remain fundamentally susceptible to this limitation. Future research should further address this concern through: (1) Ensuring complete anonymity via computer-administered surveys to alleviate evaluation apprehension; (2) Employing indirect measurement techniques (e.g., Implicit Association Tests or behavioral paradigms) as alternatives to explicit self-reports; (3) Incorporating established social desirability scales (e.g., Marlowe-Crowne or Balanced Inventory of Desirable Responding) as statistical covariates during analysis; and (4) Adopting mixed-method designs that triangulate quantitative survey data with qualitative interviews or observational validation. Collectively, these methodological refinements would enhance measurement validity by accounting for both conscious impression management and unconscious response biases inherent in socially sensitive self-reported attitudinal or behavioral data.

## Conclusion

This study examined how observers’ perceptions and reactions to sexual harassment are influenced by multiple factors, including harassment type, observer gender, and individual characteristics. Results demonstrated that these factors significantly affected observers’ evaluations, emotional responses, attributions of blame, and levels of sympathy toward victims. Furthermore, the analysis revealed two opposing belief-value systems shaping observers’ responses: a positive system reflecting respect and concern for victims’ rights and dignity, and a negative system reflecting disregard and indifference for victims’ rights and dignity. These findings suggest that effective interventions to combat sexual harassment should target these underlying cognitive frameworks to foster more empathic and supportive attitudes toward victims.

## Data Availability

The raw data supporting the conclusions of this article will be made available by the authors, without undue reservation.
