# Supplementary material for: Experimental study of factors influencing observers’ perceptions and reactions to sexual harassment in Chinese university students
Source: Front Psychol. 2025 Aug 14;16:1525006. doi: 10.3389/fpsyg.2025.1525006 (PMC12391118; doi:10.3389/fpsyg.2025.1525006)
Supplement: Supplementary file 1 [file Table_1.docx]

*Supplementary Results*

**Experimental Study of Factors Influencing Observers' Perceptions and Reactions to Sexual Harassment in Chinese University Students**

Zien Huang^1#^，Jialuo Lai^1#^，Fei Xin^1*^

^#^These authors contributed equally to this work.

**Affiliations**

^1^School of Psychology, Shenzhen University, Shenzhen, China

***Corresponding author:**

Fei Xin

School of Psychology, Shenzhen University, Nanhai Ave 3688, Shenzhen 518060, Guangdong, China.

Tel/Fax: 86-755-86581067

Email: [feixin.fx@gmail.com](mailto:feixin.fx@gmail.com)

**The file includes:**

Tables S1

| **Table S1.** Comparison of Repeated Measures ANOVA and Generalized Estimating Equations (GEE). | | | | | | |
| --- | --- | --- | --- | --- | --- | --- |
| **Variable** | **Main Effects (Harassment Type)** | | **Main Effects (Gender)** | | **Interaction** | |
|  | *F* [2,66]/$\chi^{2}\left( 2 \right)$ | *p* | *F* [1,67]/$\chi^{2}\left( 1 \right)$ | *p* | *F* [2,66]/$\chi^{2}\left( 2 \right)$ | *p* |
| **Valence** | 121.016/173.680 | < 0.001/< 0.001 | 5.914/6.956 | 0.018/0.008 | 0.912/1.539 | 0.404/0.463 |
| **Arousal** | 55.396/117.774 | < 0.001/< 0.001 | 2.794/2.791 | 0.099/0.095 | 1.156/2.692 | 0.321/0.260 |
| **Perception** | 329.553/699.041 | < 0.001/< 0.001 | 0.737/1.318 | 0.394/0.251 | 2.707/5.771 | 0.074/0.056 |
| **Victim Sympathy** | 59.419/120.654 | < 0.001/< 0.001 | 5.278/6.254 | 0.025/0.012 | 2.394/3.216 | 0.099/0.200 |
| **Victim Blaming** | 5.578/6.040 | 0.005/0.049 | 5.778/7.101 | 0.019/0.008 | 2.484/1.620 | 0.087/0.445 |
| The expression format for statistics and *p*-values is: Repeated Measures ANOVA/Generalized Estimating Equations. | | | | | | |
